# 3-Year follow-up of a single magnetically controlled growing rod with contralateral gliding system and apical control for early onset scoliosis

**DOI:** 10.1007/s43390-020-00098-1

**Published:** 2020-03-30

**Authors:** Sebastiaan P. J. Wijdicks, Simon Toftgaard Skov, Haisheng Li, René M. Castelein, Moyo C. Kruyt, Cody Bünger

**Affiliations:** 1 Department of Orthopaedic Surgery, University Medical Center Utrecht, Heidelberglaan 100, 3584CX Utrecht, The Netherlands; 2 Department of Orthopaedic Surgery, Aarhus University Hospital, Palle Juul-Jensens Boulevard 99, 8200 Aarhus, Denmark; 3 Orthopaedic Research Laboratory, Aarhus University Hospital, Palle Juul-Jensens Boulevard 99, 8200 Aarhus, Denmark

**Keywords:** MCGR, Early onset scoliosis, Growing rod instrumentation, Growth friendly system, Minimal invasive surgical procedure, Apical control

## Abstract

**Study design:**

Two-center retrospective cohort study.

**Objective:**

The aim of this study is to investigate the clinical effectiveness and safety of the MCGR hybrid in terms of spinal growth, 3D correction, balance, and complications.

**Summary of background data:**

The magnetic-controlled growing-rod (MCGR) growth instrumentation method has gained popularity for early onset scoliosis (EOS) treatment in the past years due to the non-invasiveness of the subsequent interval elongation procedures. To improve 3D correction and reduce the costs, we combined a single concave MCGR with a sliding rod on the convex side to control the apex.

**Methods:**

A retrospective cohort study of 18 EOS children with an average 3-year follow-up (range 2.0–3.7) from two European spine centers treated with the single MCGR hybrid concept; 14 primary and 4 conversion cases. The primary and conversion cases were both evaluated preoperatively, postoperatively, 1 year, 2 years, and last follow-up.

**Results:**

Mean age was 9.9 (SD ± 2.9 years). The average frontal Cobb angle was reduced from mean 65° to 30° postoperatively, and had increased to 37° at latest follow-up. Rotation of the apical vertebra improved from mean 27° to 20° postoperatively which was partially lost to 23°. Kyphosis and lordosis both increased by an average of 5° during the time of follow-up. Spinal balance was improved. The post-implantation T1–S1 spine growth rate averaged 10 mm/year at last follow-up. There were 13 implant-related complications in 6 out of 18 patients. No screw pull-outs and nor surgical site infections were registered.

**Conclusions:**

This is the first medium-term results of a single MCGR hybrid construct. Maintenance of correction and growth are reasonable, and the complication rate is relatively low as compared to bilateral MCGR application.

**Level of evidence:**

III.

## Introduction

Progressive early onset scoliosis (EOS) can become a hazard to pulmonary development and function [1, 2]. Different “growth-friendly systems” and implants have been developed to control the scoliosis deformity and allow for continuous spinal growth and thereby support the truncal development. Traditional growing rods were widely used in severe EOS throughout the last decades, but required repeated surgical lengthening procedures under general anesthesia coupled with relatively high infection rates [3, 4]. Magnetically controlled growing rods (MCGRs) (Magec, NuVasive, San Diego, CA, USA) were introduced about 10 years ago with the first publication in 2012 [5]. It is recommended for dual or single rod application according to the needs of the individual patient. MCGRs allow for non-invasive distraction of the rod construct by electromagnetic stimulation without sedation.

We combined a single MCGR to drive the concave lengthening with a contralateral passive sliding-rod construct on the convexity to improve the 3D deformity correction. An anchor site was added at the apex to increase the stability and aid for axial deformity correction [6]. Dual rods instead of single rods have been advised in the traditional growing-rod treatment because of better correction, spinal growth, and lower implant-related complications [7, 8]. After MCGR became available, the dual MCGR rod construct has become a popular treatment in many centers despite the high initial implant cost [5]. The advantages of a dual MCGR over a single MCGR construct has been advocated by a recent systematic review which found fewer implant-related failures including a lower frequency of rod breakage [7]. The bilateral support of our proposed hybrid construct follows a dual-rod principle with added apical support and could reduce complications including rod breakage. Finally, costs using the MCGR can be reduced by obviating the need for surgical distractions and only requiring out-patient clinic visits [9]. Moreover, reducing the initial device costs of a dual MCGR (20.000 USD) to a single MCGR (10.000 USD) could further reduce overall costs [9–11]. Substituting the second MCGR with an inexpensive gliding construct anchored apically with one or two pedicle screws could further reduce overall costs. Therefore, utilizing a single MCGR in this hybrid concept may improve 3D correction and spinal balance at a reduced cost.

The aim of this study was to investigate both the 3D correction, the spinal growth and the complication rate of our new hybrid growing-rod–sliding-rod concept. We report the combined results from two European scoliosis centers: U and A.

## Materials and methods

### Study design

A retrospective cohort study of all consecutive EOS patients irrespective etiology, treated with the hybrid MCGR and apical control from September 2014 to June 2016 at U and A spine centers was evaluated. The inclusion criteria for this study and MCGR hybrid surgery were: skeletal immaturity, progressive scoliosis, and a major curve of more than 45°. A sample size of 18 was attained by selecting all patients who received the hybrid MCGR at any time at one of the two institutions and had a minimum of 2-year follow-up. We report preoperative, postoperative, 6 months, 1 year, 2 years, and last follow-up results including spinal growth, 3D correction, complications, and re-operations. Data collection and data storage were approved in accordance with the national guidelines for research ethics and data protection. This study followed the STROBE guideline for reporting observational studies [12].

### Surgical techniques

The patients were placed in balanced prone position without traction. Standard infection prevention precautions were taken including perioperative intravenous antibiotics. Topical wound administration (e.g., Vancomycin) was not applied in any of the patients. A posterior midline approach was used at the three strategic anchor sites for pedicle screw placement, identified by fluoroscopy. Commercially available pediatric spine implants for 4.5- or 5.5-mm rods including MCGR were used. On the concave side, a contoured MCGR was tunneled subfascially and mounted at the proximal and distal anchor sites. On the convex side, a unilateral pedicle screw anchor site was added at the apex and used to mount the pre-assembled contoured passive sliding-rod construct, bridging the intermediate unexposed segments of the spine. The apex of the spine was identified intra-operatively and approached with a separate incision. The rods were contoured before insertion to accommodate proximal kyphosis and distal lordosis, to facilitate deformity correction, and to avoid proximal and distal junctional failures. Obviously, the actuator of the MCGR remains problematic also in this hybrid strategy. However, since the MCGR is positioned in the concavity, there is more margin for positioning without prominence as this side is typically deeper. On the more superficial convex side, the sliding rod can be contoured in any shape which is an advantage.

**Fig. 1:**
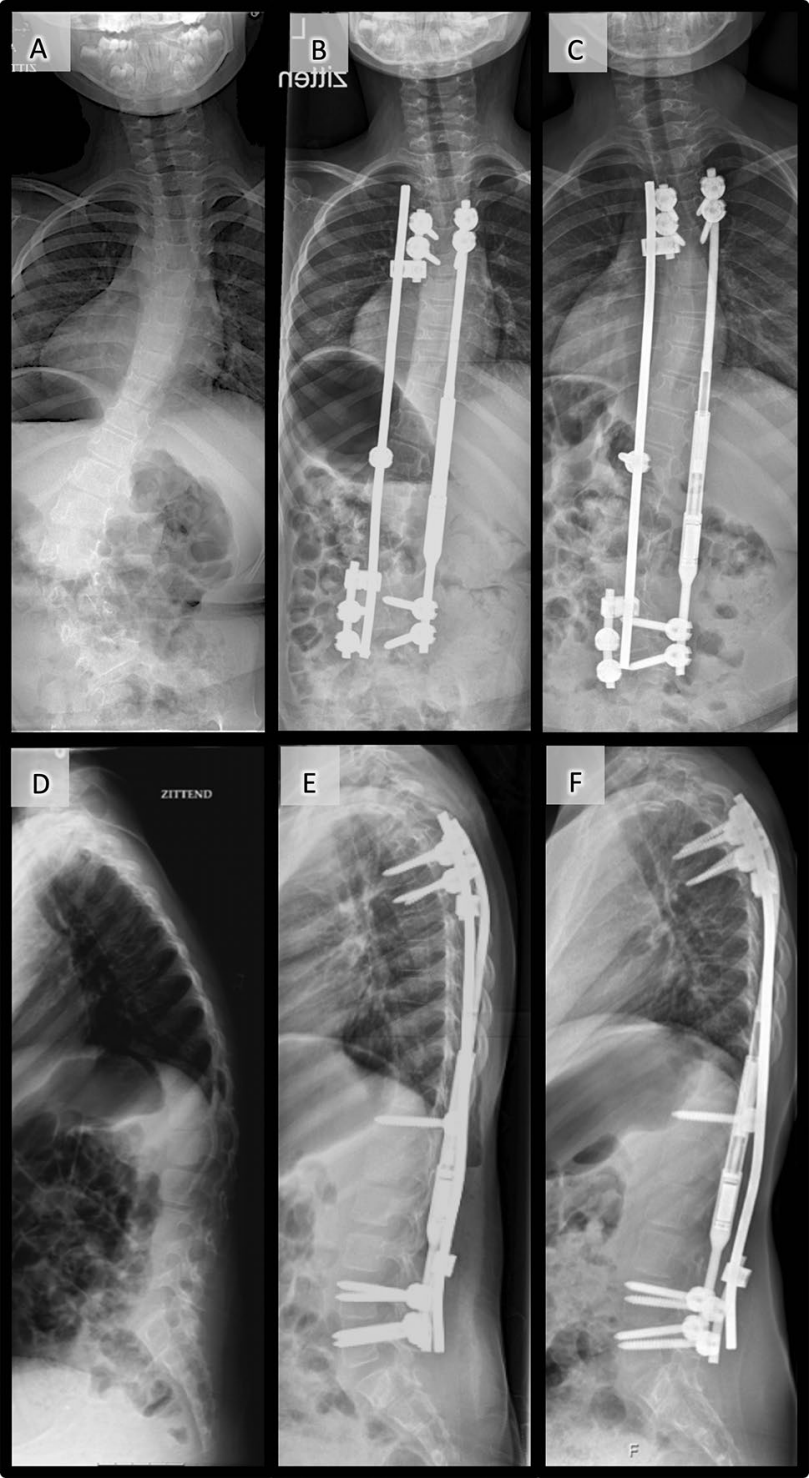
Combined single magnetic rod and parallel block sliding rod system in a 7-year-old girl with spinal muscular atrophy type 2: **a** frontal radiographs made preoperatively, **b** postoperative, and **c** at the time of final follow-up, and **d** sagittal radiographs made preoperatively, **e** postoperative, and **f** at the time of last follow-up

**Fig. 2:**
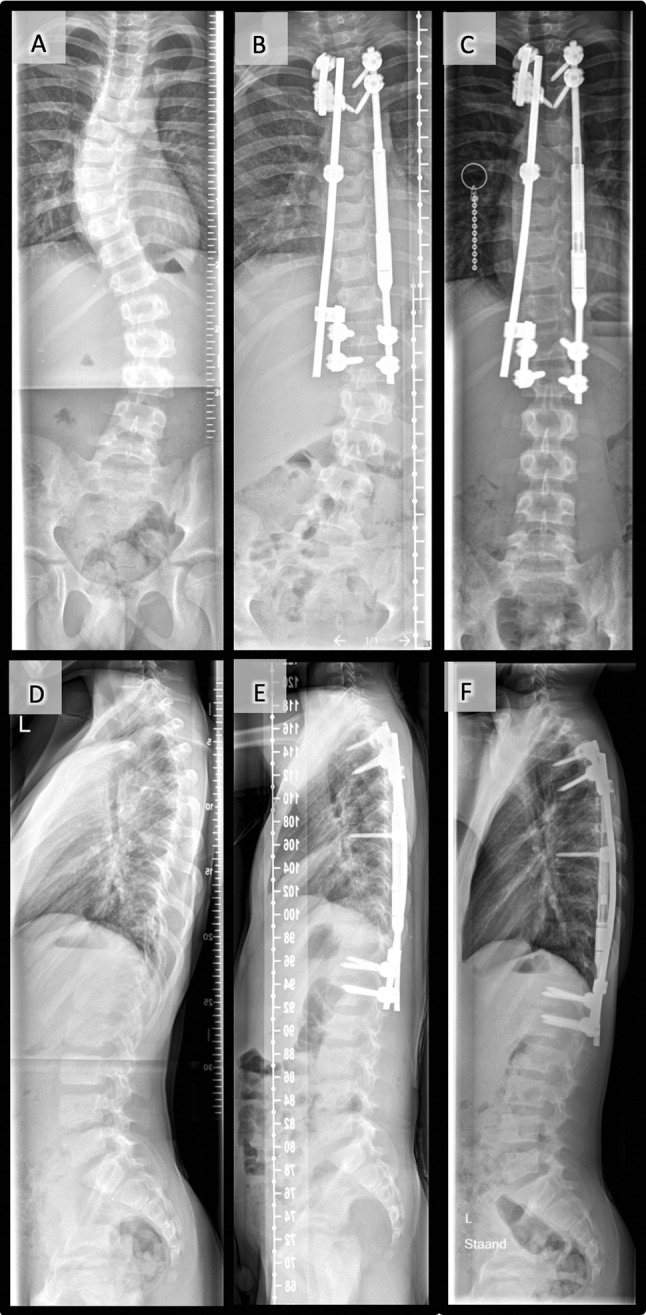
Combined single magnetic rod and parallel block sliding rod system in an 8-year-old girl with idiopathic scoliosis: frontal radiographs made **a** preoperative, **b** postoperative, and **c** at the time of final follow-up. Sagittal radiographs made **d** preoperative, **e** postoperative, and **f** at the time of last follow-up

**Fig. 3:**
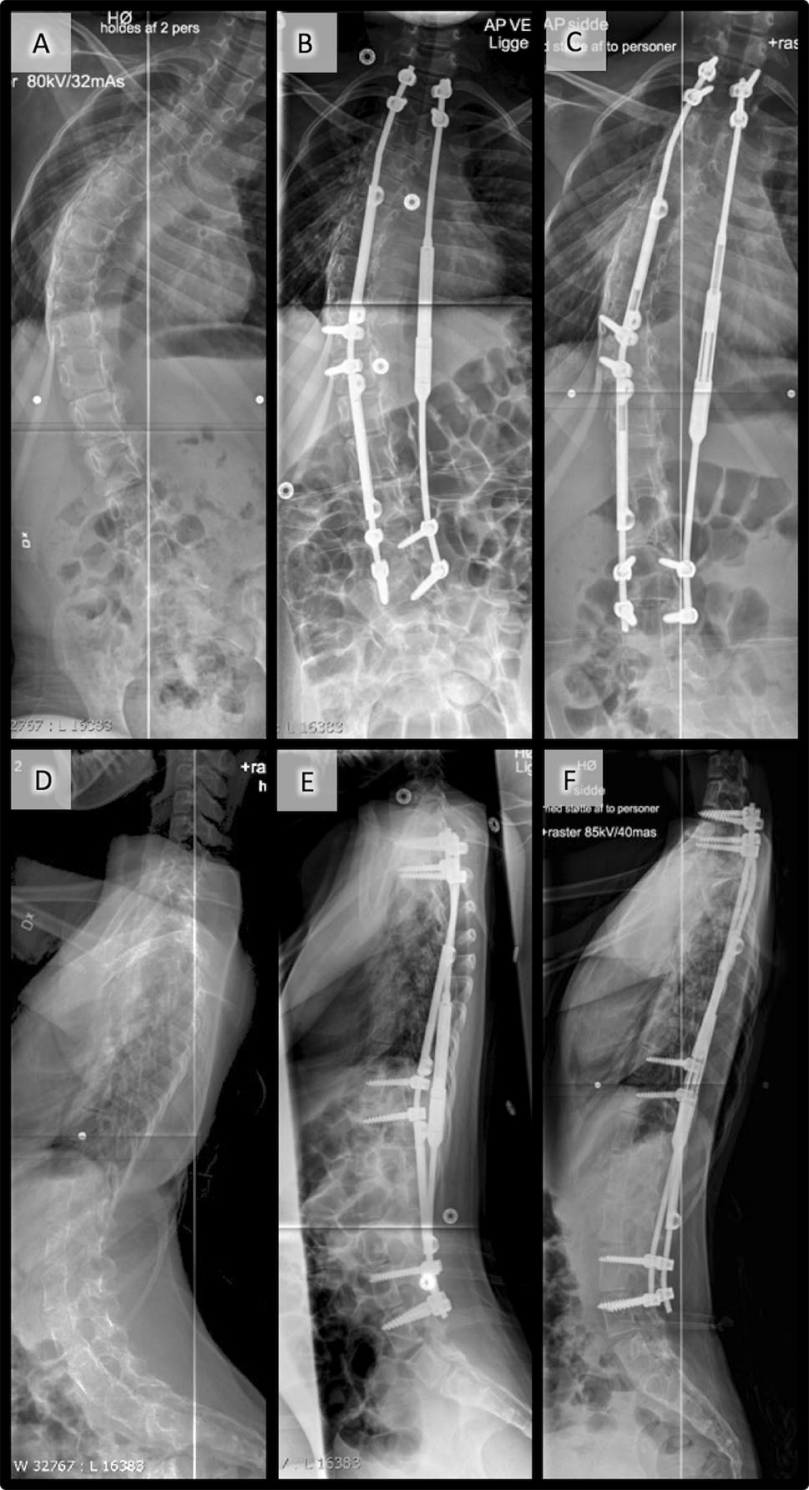
Combined single magnetic rod and CB system in an 11-year-old girl with cerebral palsy: **a** frontal radiographs made preoperatively, **b** postoperative, and **c** at the time of final follow-up, and **d** sagittal radiographs made preoperatively, **e** postoperative, and **f** at the time of last follow-up

The passive sliding construct on the convex side differed between our two centers. In U, the 5.5 mm K2M Mesa® and Magec® systems were used. One long apical anchored rod was allowed to slide through proximal and distal parallel connectors. The parallel connectors had an oversize hole at the sliding-rod connection which was left open without set-screw (5.5 mm-diameter hole for a 4.5 mm rod) (Figs. 1 and 2). In A, the Xia® and K2M Mesa® 4.5 or 5.5 CD Legacy® system and Magec 4.5 or 5.5 rods were used. The CB system was used for the convex side; three 4.5 mm rods assembled with two longitudinal connectors as growth tubes; each unlocked at one end to enable passive sliding between the three anchoring sites (Fig. 3).

MCGR distraction by external magnetic stimulation was conducted on an out-patient basis at 2.5–3-month intervals based on the manufacturer instructions [13]. Biplanar scoliosis radiographs were taken postoperatively and at 6-month intervals to balance between radiation exposure and adequate follow-up of the MCGR. Failure to distract was defined as a combination of multiple instances of slippage of the MCGR’s internal mechanism (resulting in an audible clunking sound and failure of the internal magnet to distract the MCGR) and a lack of any MCGR distraction on consecutive radiographs.

### Data collection

**Fig. 4:**
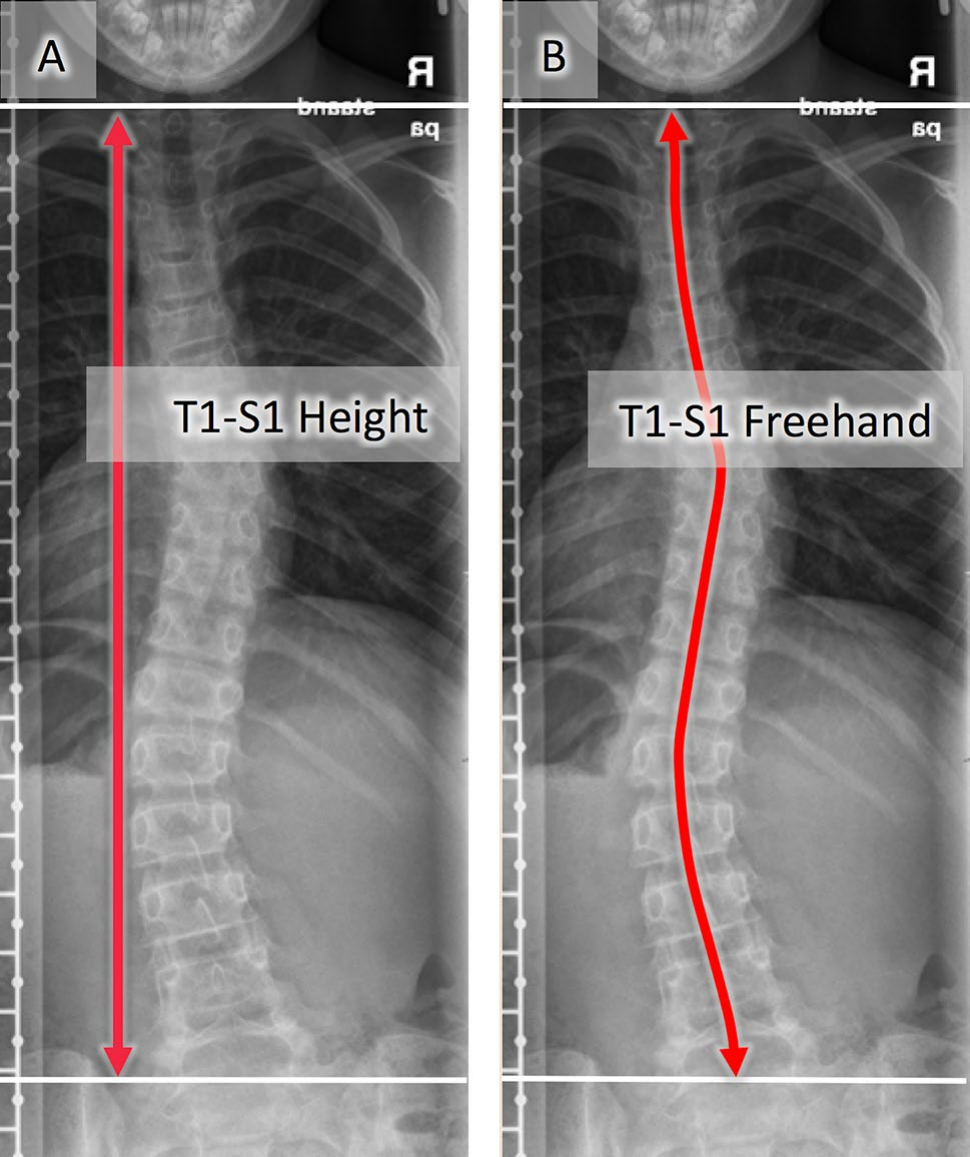
T1–S1 spinal length measurements: T1–S1 height measurement example (Fig. 1a) and T1–S1 freehand example (Fig. 1b)

Electronic patient files were reviewed for complications, re-operations, and distraction failures of the MCGR. Digital biplanar scoliosis radiographs were evaluated using Surgimap 2.2.14 spine measurement software (Nemaris Inc, New York, NY, USA). An investigator from each center performed the measurements. To reduce potential bias, the numbers were cross-audited and eventual discrepancies were solved with consensus. Scoliosis Cobb angle as well as the kyphosis (T4–T12) and lordosis (L1–L5) angles were measured. The rotation of the apical vertebra was measured according to the Nash–Moe method, because neither one of our centers have ultra-low-dose 3D-imaging (e.g., EOS3D) available, and CT imaging is only applied on clinical indication to minimize the radiation exposure [14]. MCGR actuator diameter (narrow part 9.02 mm, wide part 10.50 mm) was applied to calibrate the radiographs for height measurements. T1–T12 height, T1–S1 height, and instrumented height were measured as the perpendicular distance between horizontal lines through the midpoints of the chosen vertebral endplates on coronal radiographs (Fig. 4a). The T1–S1 freehand measures represent a spinal length with a line drawn through the exact midpoint of the upper and lower endplates of every vertebra resulting in a line following the contour of the spine to achieve a more precise spinal length measurement. The Surgimap Free Rod tool was used for this measure by trailing the center points of the vertebral body endplates (Fig. 4b). The distraction length was measured on the MCGR. Growth rates were calculated based on the measurements from the first postoperative radiographs to the time point of the respective follow-ups. Furthermore, apical translation, coronal balance, and sagittal balance (in ambulatory patients) were measured to assess whether the deformity correction affected the global balance of the spine.

### Statistics

Descriptive statistics and statistical analysis were performed with IBM SPSS Statistics 24.0 (IBM Corp. Armonk, New York, NY, USA) with a level of significance of *p* < 0.05. Postoperative and last follow-up outcomes were analyzed with paired *t* tests. Wilcoxon signed-rank test was used for data appearing non-normally distributed.

## Results

### Patient demographics

A total of 18 patients were included, followed up, and analyzed; 9 patients from each center, including 14 primary cases, and 4 conversion cases from traditional growing rods (Table 1). Of the 14 primary cases: 10 patients failed initial brace treatment, and 4 had curves not suited for brace treatment. All the patients were skeletally immature, mean age 9.9 ± 2.9 years (range 6–18) with the oldest patient having a bone age of 11 years. All had a progressive scoliosis and an average Cobb angle of 65° ± 12° (range 46–86). The Cobb angle reduction of the primary cases was 57% and the Cobb angle for the conversion cases was 7%, compared to the initial curve. The conversion surgery itself yielded little extra correction, because the spines were stiff or had partial support by the previous system applied. The etiology was 50% neuromuscular (*n* = 9), 33% idiopathic (*n* = 6), and 17% syndromic (*n* = 3). Mean overall surgery time for MCGR implantation procedure was 193 min (range 96–278) and their average hospital admission time was 5.4 days (range 1–12). The average follow-up time was 3 years (range 2–3.7).

**Table 1:** Patient demographics

	All (*n* = 18)	U (*n* = 9)	A (*n* = 9)
No. of patients (male:female)	4:14	3:6	1:8
MCGR case (primary:conversion)	14:4	8:1	6:3
Etiology (Neuromuscular:Idiopathic:Syndromic)	9:6:3	4:4:1	5:2:2
Age at the time of MCGR surgery (year)	9.9 (6.4–18.1)	8.0 (6.4–9.3)	11.7 (6.9–18.1)^a^
Surgery time (min)	194 (96–278)	200 (135–278)	187 (96–260)
Days of admission (days)	5.4 (1–12)	6.4 (3–12)	4.3 (1–7)
Instrumented levels (no. of levels)	14 (11–16)	13 (11–16)	14 (12–16)
Postoperative FU from MCGR surgery (mos)	37 (26–47)	38 (29–47)	35 (26–47)

^a^Skeletally immature, 5–7 years delayed according to hand bone age

### Radiographic outcomes

**Fig. 5:**
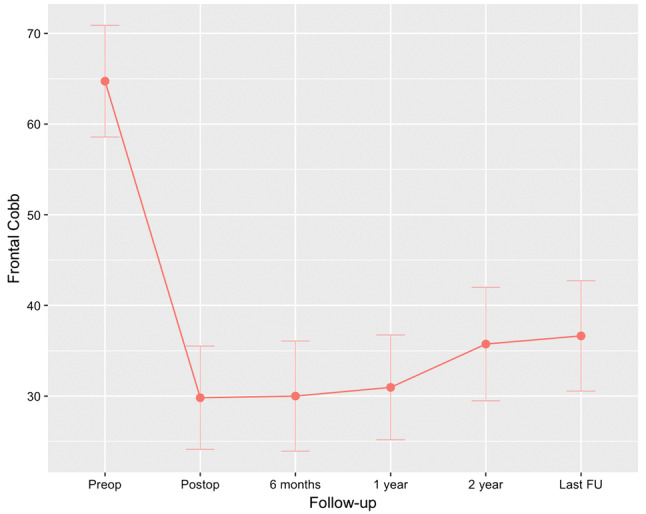
Frontal Cobb Angle: error bars represent the 95% confidence intervals

**Fig. 6:**
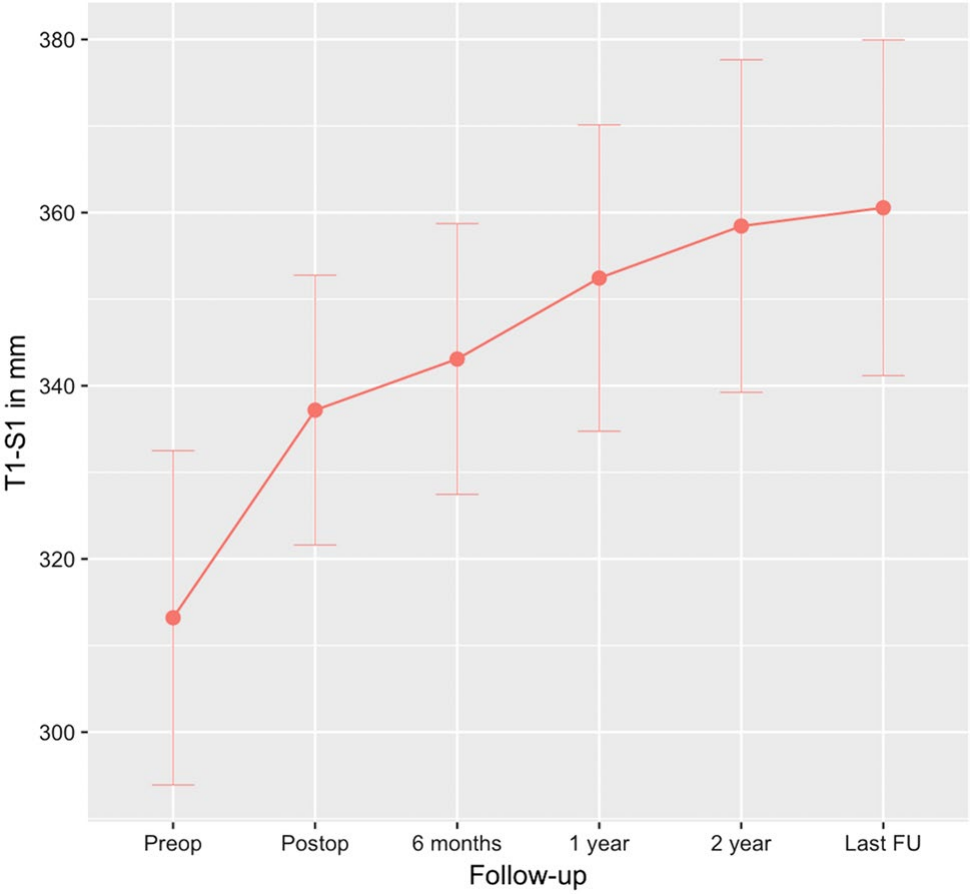
T1–S1 height: error bars represent the 95% confidence intervals

The Cobb angle of the primary cases changed from mean 65° ± 13° preoperatively to 28° ± 12° postoperatively (55% reduction). The mean frontal Cobb angle of the conversion cases was 64° ± 11° at fist surgery and 38° ± 9° at conversion. This curve was reduced considerably less as expected to 35° ± 6° (Table 2). For all cases (primary and conversions) reduction from initial until after conversion was 59° ± 17° to 30° ± 11° (Table 3). This angle slightly increased to 37° ± 12° at latest follow-up, *p* < 0.001 (95% CI 3.3–10.3) (Fig. 5). Individual demographics for every patient are visible in Table 2. Rotation of the apical vertebra improved from mean 27° ± 8° to 20° ± 9° postoperatively, but was partially lost to 23° ± 9° during follow-up, *p* = 0.261 (95% CI − 2.5–8.6). Kyphosis and lordosis both increased by an average of 5° during follow-up (Table 3). T1–S1 height increased from average 337 ± 31 mm postoperatively to 361 ± 39 mm at last follow-up, *p* < 0.001 (95% CI 13.5–33.3) (Fig. 6). Spinal T1–S1 growth rate averaged 10 mm/year over 3 years until last follow-up (Table 4). There was no difference in growth rate between conversion and primary cases (Table 2). None of the patients reached the maximum distraction point of the rod during follow-up.

**Table 2:** Primary vs. conversion: angle and spinal growth rates (mean ± SD; range)

	Pre-op major curve (*n* = 18) (deg)	Post-op major curve (*n* = 18) (deg)	Last FU major curve (*n* = 18) (deg)	T1–T12 length gain rate post-op to last FU (*n* = 18) (mm/year)	T1–S1 length gain rate post-op to last FU (*n* = 18) (mm/year)	Instrumented gain rate post-op to last FU (*n* = 18) (mm/year)
Primary	65 ± 13 (46–86)	28 ± 12 (8–49)	38 ± 14 (19–67)	6.1 ± 5.6 (− 3.6–19.3)	10.2 ± 9.2 (− 0.3–30.3)	9.1 ± 7.2 (− 0.4–21.4)
Conversion	38 ± 9 (26–47)	35 ± 6 (29–43)	33 ± 5 (28–39)	8.8 ± 4.4 (5.6–15.3)	10.5 ± 6.4 (4.7–19.5)	8.9 ± 5.4 (.04–16.2)

*Pre-op* radiograph before MCGR implantation surgery, *Post-op* radiograph before discharge from hospital, *FU* follow-up

**Table 3:** 3D correction: angle, rotation, or distance (mean ± SD; range)

	Pre-op (*n* = 18)	Post-op (*n* = 18)	Last FU (*n* = 18)	Change pre-op to post-op (*n* = 18)	Change post-op to 2-year FU (*n* = 18)	Change post-op to last FU (*n* = 18)
Frontal Cobb (deg)	65 ± 12* (46–86)	30 ± 11 (8–49)	37 ± 12 (19–67)	− 35 ± 12 (− 16–65)	6 ± 7 (− 4–18)	7 ± 7 (− 4–18)
Rotation Nash–Moe (deg)	27 ± 8 (13–42)	20 ± 9 (5–36)	23 ± 9 (6–41)	− 7 ± 9 (− 26–11)	1 ± 10 (− 15–22)	3 ± 11 (− 15–26)
Apical translation (cm)	5.5 ± 2.7 (1.5–11.1)	2.7 ± 1.6 (0.1–5.5)	2.8 ± 1.6 (0.3–6.0)	− 2.8 ± 2.2 (− 8.1–0.2)	0.1 ± 1.6 (− 2.5–3.9)	0.1 ± 1.8 (− 2.7–3.9)
Coronal balance (cm)	2.2 ± 1.4 (0.3–5.5)	1.9 ± 1.8 (0.1–6.5)	1.5 ± 1.6 (0.1–5.6)	− 0.2 ± 2.1 (− 3.5–4.2)	− 0.3 ± 2.7 (− 4.6–6.3)	− 0.4 ± 2.3 (− 4.0–6.4)
Kyphosis T4–T12 (deg)	27 ± 19 (2–67)	20 ± 12 (4–53)	24 ± 17 (0–62)	− 7 ± 15 (− 47–13)	3 ± 11 (− 21–29)	5 ± 11 (− 21–29)
Lordosis L1–L5 (deg)	37 ± 17 (6–65)	34 ± 13 (17–57)	40 ± 13 (13–64)	− 3 ± 12 (− 26–15)	5 ± 9 (− 8–26)	5 ± 10 (− 8–31)
Sagittal balance^†^ (cm)	3.7 ± 2.0 (0.0–6.3)	4.0 ± 2.6 (0.0–9.6)	3.1 ± 2.4 (0.2–7.9)	− 0.4 ± 2.4 (− 2.8–3.9)	− 1.5 ± 3.4 (− 7.3–5.6)	− 0.9 ± 3.9 (− 8.5–5.6)

*Pre-op* radiograph before MCGR implantation surgery, *Post-op* radiograph before discharge from hospital, *FU* follow-up

*Pre-primary values applied for all conversion cases (59° ± 17° if values before magnetic rod implantation)

^†^Only in ambulatory patients

**Table 4:** Height measurements and spinal growth rates (mean ± SD; range)

	Pre-op (*n* = 18) (mm)	Post-op (*n* = 18) (mm)	Last FU (*n* = 18) (mm)	Length gain rate post-op to 1-year FU (*n* = 18) (mm/year)	Length gain rate post-op to 2-year FU (*n* = 18) (mm/year)	Length gain rate post-op to last FU (*n* = 18) (mm/year)
T1–S1	313 ± 39 (270–387)	337 ± 31 (304–392)	361 ± 39 (313–449)	13.2 ± 12.5 (− 7.0–30.3)	11.2 ± 9.4 (− 6.6–30.3)	10.3 ± 8.5 (− 0.3–30.3)
T1–T12	196 ± 22 (165–237)	208 ± 17 (187–242)	223 ± 22 (185–278)	9.0 ± 7.2 (− 1.9–21.7)	7.5 ± 5.5 (− 1.1–19.3)	6.7 ± 5.4 (− 3.6–19.3)
Instrumented	239 ± 40 (173–308)	259 ± 39 (189–340)	281 ± 46 (199–364)	14.0 ± 10.7 (− 9.0–37.3)	9.6 ± 9.6 (− 15.3–29.9)	9.1 ± 6.7 (− 0.4–21.4)
Freehand T1–S1 coronal		352 ± 33 (312–404)	375 ± 41 (320–475)	11.1 ± 15.8 (− 20.5–39.4)	10.8 ± 11.5 (− 6.9–37.3)	10.2 ± 10.1 (0.2–37.3)
Freehand T1–S1 sagittal		355 ± 31 (310–405)	379 ± 40 (316–448)	12.3 ± 13.0 (− 16.2–31.8)	11.0 ± 8.1 (− 1.3–29.1)	10.3 ± 7.5 (− 1.3–23.8)

*Pre-op *radiograph before MCGR implantation, *Post-op* radiograph before discharge from hospital, *FU* follow-up

The average apical translation (deviation from the midline) was 5.5 ± 2.7 cm and improved to 2.7 ± 1.6 cm and remained stable at 2.8 ± 1.6 cm at last follow-up. Coronal balance (deviation of the C7 plumb line from the sacral midline) changed 2.2 ± 1.4 cm to 1.9 ± 1.8 cm postoperatively to 1.5 ± 1.6 cm at last follow-up. The sagittal balance in ambulatory patients (*n* = 12) changed from 3.7 ± 2.0 cm to 4.0 ± 2.6 cm postoperatively and to 3.1 ± 2.4 cm at last follow-up (Table 5).

### Complications

No intraoperative or perioperative procedure-related adverse events were registered. Five unplanned surgeries occurred in 4 out of 18 patients (22%). There was a total of 9 implant-related complications in 6 out of 18 patients (33%) (Table 5). In four patients, the system was converted to a different growing-rod construct. There were four nonsurgical complications. Detailed overview of complications is visible in Table 2. No superficial or deep infections or material failures (e.g., screw pull out) were experienced. We did not see obvious PJK (> 10°) at the final follow-up. The average implant-related complication rate of our merged data was 0.18 per patient per year. There was a non-significant difference between the average weight of patients with and without complication, 34 kg versus 29 kg, *p* < 0.312 (95% CI − 5.3–15.6), respectively. During the revision surgery, metallosis was found at the actuator to rod junction. During revision, we did not observe obvious fusions. We did not exchange the MCGR with a new MCGR, the MCGRs that were removed typically failed to distract when removed.

**Table 5:** Demographics; individual details and complications

#	Age (year)	Sex	Surgery before MCGR	Scoliosis type	Weight before surgery (kg)	MCGR size (mm)	MCGR distraction (mm)	Pre-pri-marymajor curve (deg)	Pre-op mcgr major curve (deg)	Post-op mcgr major curve (deg)	Last FU major curve (deg)	Complications
U1	7.5	F	No	NM	21	5.5	26	–	54	27	37	–
U2	8.2	M	No	Idio	28	5.5	14	–	47	10	22	–
U3	6.7	F	No	NM	25	5.5	19	–	80	15	19	–
U4	9.0	F	No	NM	18	5.5	15	–	76	40	41	–
U5	8.7	F	Yes	Syn	22	4.5	30	60	42	43	39	#1) MCGR rod breakage between the actuator and distal fixation after 2.5 years. Revised to other growth friendly system
U6	9.3	M	No	Idio	32	5.5	12	–	86	49	67	#1) Distraction failure after 2.0 years. Asymptomatic and currently without curve progression. Watchful waiting†
U7	8.8	M	No	NM	25	5.5	14	–	68	48	61	–
U8	7.2	F	No	Idio	37	5.5	6	–	54	24	41	#1) Distraction failure. Distraction under general anesthesia unsuccessful and MCGR and tested defective after removal. Revised after 2.1 years to other growth-friendly system. †
U9	6.4	F	No	Idio	21	5.5	14	–	58	24	35	–
A1	6.9	F	No	NM	16	4.5	22	–	73	27	27	–
A2	9.8	F	Yes	Idio	36	4.5	31	60	26	29	30	#1) Distraction failure after 2.1 years. Resolved with distraction under general anesthesia^†^ #2) Back pain and clinically reduced distractions after 2.9 years. Hand bone age was 1.5 older than her chronologic age. Final fusion performed instead of revision
A3	10.2	F	No	NM	40	5.5	21	–	84	29	44	–
A4	18.1*	F	Yes	NM	32	5.5	22	80	47	31	28	–
A5	11.7	F	No	NM	50	5.5	9	–	64	31	37	#1) Distraction of < 2 mm during the first 2 lengthenings. MCGR distraction achieved at 9 months and at subsequent lengthenings (no surgery) #2) Progressive trunk shift after 1.6 years solved by conversion of the caudal passive sliding construct to TGR principle. ‡
A6	12.2	F	No	Syn	25	4.5	6	–	46	8	22	#1) Progressive trunk shift due to secondary lumbar curve progression 5 months post-op. ‡ Corrected by conversion to TGR principle of the sliding rod and 4 subsequent surgical distractions #2) Sustained Th1 + Th2 fracture (AO type A1) after falling from a standing height 2 month after trunk shift revision. Not related to the scoliosis treatment. Treated conservatively with cervicothoracic bracing. No sequelae
A7	12.6	F	No	NM	27	4.5	30	–	63	35	37	–
A8	12.9	M	Yes	Syn	43	5.5	15	57	37	35	36	–
A9	11.1	F	No	Idio	46	5.5	20	–	57	32	38	–

*Age* at time of primary MCGR implantation, *Pre-Primary major curve* major curve before any scoliosis surgery, *FU* follow-up, *M* male, *F* female, *NM* neuromuscular scoliosis, *Syn* syndromic scoliosis, *Idio* idiopathic scoliosis

*Skeletally immature, 5–7 years delayed according to hand bone age

^†^Distraction failure: combination of multiple instances of slippage of the MCGR’s internal mechanism (resulting in an audible clunking sound and failure of the internal magnet to distract the MCGR) and a lack of any MCGR distraction on consecutive radiographs

^‡^Trunk shift: lateral deviation of the center of the trunk

## Discussion

These medium-term follow-up results suggest that a combination of a single concave MCGR and a contralateral passive sliding system with apical control is feasible and safe. The MCGR Hybrid was able to correct and maintain alignment and growth comparable to other MCGR results [15–18]. The observed slight increase of the major curve is a well-known phenomenon that has been observed with all other growth-friendly systems including MCGR, as well [3, 16–18]. This slight loss of correction over time did not mandate revision in any of the patients. Rotation in the apex after surgery did not change significantly over time. The minimal increase in rotation could indicate that this hybrid has apical control, although this assessment on plain X rays is relatively inaccurate. The mean T1S1 growth rate of 10 mm/year over 3 years is well acceptable and in accordance to physiological growth [19, 20]. In general, we have the experience that conversion patients have a stiffer spine. This is not obvious in terms of growth from our results. Growth is better than the 3–4 mm/year reported in MCGR papers with similar follow-up time [17, 18]. Sankar et al. investigated length gain achieved with every distraction of TGR [21]. They found that length gains decreased with every additional lengthening over time. More recently, Cheung et al. found similar reduced lengthenings with MCGR [22]. We found higher distraction rates in the first-year results (13 mm/year) compared to the distraction rate in the period from 1 year until last follow (7 mm/year distraction). This supports our general impression of diminishing returns with the MCGR over time [21, 22]. Whether our protocol of distraction every 3 months up to stalling of the actuator is the most optimal to prevent diminishing returns remains to be determined. Other publications have done this on a monthly basis or semi-annually [21, 22].

The MCGR that were removed typically failed to distract and were send for analysis to the manufacturer. Unfortunately, this did not give more insight in the failure mechanism. The sliding construct did not show failures.

Currently, no publication has reported on spinal balance outcomes after MCGR surgery. Akbarnia et al. investigated balance in a group of the traditional growing-rod EOS patients with a comparable minimum 2-year follow-up time [23]. The coronal balance (deviation from the midline) with growing rods changed from 2.8 to 1.8 cm and was 2.0 cm at the latest follow-up or post-final fusion. The sagittal balance in growing rods (C7 plumb line deviation) changed from 3.7 to 2.3 cm after surgery to 3.9 cm at last follow-up [23]. If we compare our MCGR results with the traditional growing rods, we find that the coronal balance was comparable and that the sagittal balance in our group did improve (0.6 cm). While encouraging, our group is too small to conclude a benefit in balance from the hybrid system.

Growth-friendly surgeries have high rates of both planned and unplanned surgeries because of surgical lengthening’s and complications, respectively [4]. MCGR has reduced planned re-operations by shifting from surgical to nonsurgical lengthening. However, unplanned surgeries because of complications do occur. MCGR studies with a minimum 2-year follow-up report that patients requiring unplanned surgery ranged from 39 to 75%. 92 patients combined from four studies experienced 17 cases of a nonfunctioning MCGR and 12 cases of rod fracture requiring unplanned surgery (a total of 31%) [15, 16, 24, 25]. Our cohort consisting of 18 patients experienced two cases of a non-distracting MCGR after implantation and one rod fracture requiring unplanned reoperation (a total of 17%). The implant-related complications (including complications not requiring unplanned surgery, e.g., temporary distraction failures or painful distractions) ranged from 48 to 75% in studies with a minimum 2-year follow-up [16, 24, 25]. One study reported a complication rate of 0.23 per patient per year [25]. Our results show a comparable or lower complication rate of 0.18 per patient per year. We found that the average time until conversion (from MCGR hybrid to other growth-friendly systems) was 1.7 years. In one patient, this reoperation was at 6 months because of a progressive trunk shift due to too high distal level of instrumentation. Whether one sliding-rod configuration should be preferred cannot be determined based on the limited data and different patient groups. Some complications were due to failure of rod distraction and it has been suggested that this is possibly due to increased body habitus (weight, height, and BMI) and increased distance between external magnet and MCGR actuator in some patients. Although our patient group is too small to draw conclusions, we did not see an obvious relation between body weight and failure. However, we did not correct for BMI.

Several studies have been published regarding cost estimates of MCGR treatment in different health care systems. The general conclusion is that the reduced number of surgeries outweighs the high initial implant cost [5, 7, 15, 16]. The results of our study, with a hybrid construct using only one MCGR, indicate performance in terms of efficacy and safety that are at least on par with dual MCGRs. Although the installation of the sliding rod on the contralateral side is a bit more challenging than an MCGR, these extra 10–20 min are probably cost-effective as the cost of the implants is reduced by about 9.000 USD. These results are far from ideal, but are currently the most optimal of all documented growth-friendly techniques. Whether the additional apical control is another advantage for 3D correction and biomechanical stability is yet to be examined.

### Limitations

The current results are at interim. Since more complications were experienced in patients with longer follow-up time, more complications are to be expected until final fusion or end of growth. Systematic errors in radiographic measurements is a potential bias (e.g., using the wrong levels in T1–S1 height). Therefore, all the outcomes were measured on five time points (postoperatively, 6 months, 1 year, 2 years, and latest follow-up) and cross-audited by two observers for discrepancies. The standard T1–S1 height measurement can increase by a reduction in major curve or kyphosis over time. To reduce this problem, we added T1–S1 freehand measurements in both coronal and sagittal planes. We included patients from two spine centers and included cases with the previous growth rod instrumentation systems who were switched to the MCGR hybrid which might be confounding the outcome of this study. However, nothing indicates that these factors (positively) influenced our results, although there may be a bias towards only inclusion of patients with an obvious dominant curve that could be treated with this strategy. We found out that for Lenke 3 or other obvious double curve configuration, this technique is probably not a good strategy. On the other hand, the patients were relatively old and some were conversion cases. In hindsight, it is arguable if the MCGR was really worth the high complication rate of this treatment for some of the patients. We believe that an age between 8 and 10 years and failed bracing is the ideal indication.

## Conclusions

This is the first report on medium-term results of a hybrid concept that consists of a single MCGR for concave distraction combined with a contralateral passive gliding rod construct with apical control. The 3D correction is good and spinal growth is preserved. The complication rate is fairly low, which suggests a cost-effectiveness as compared to dual MCGR treatment.

## Key points

- This study reported the medium-term results of 18 EOS patients treated with an MCGR hybrid of a single concave MCGR combined with a convex sliding rod with a minimum follow-up of 2 years.
- The MCGR hybrid is safe and effective in the treatment of EOS.
- The complication rate is fairly low and only uses one MCGR rod which suggests a cost-effectiveness as compared to dual MCGR treatment.

## References

[1] Branthwaite MA (1986). Cardiorespiratory consequences of unfused idiopathic scoliosis. Br J Dis Chest.

[2] Davies G, Reid L (1971). Effect of scoliosis on growth of alveoli and pulmonary arteries and on right ventricle. Arch Dis Child.

[3] Luhmann SJ, McCarthy RE (2017). A comparison of SHILLA GROWTH GUIDANCE SYSTEM and growing rods in the treatment of spinal deformity in children less than 10 years of age. J Pediatr Orthoped.

[4] Bess S, Akbarnia BA, Thompson GH (2010). Complications of growing-rod treatment for early-onset scoliosis analysis of one hundred and forty patients. J Bone Joint Surg.

[5] Cheung KMC, Cheung JPY, Samartzis D (2012). Magnetically controlled growing rods for severe spinal curvature in young children: a prospective case series. Lancet.

[6] Skov ST, Wijdicks SPJ, Bunger C (2018). Treatment of early-onset scoliosis with a hybrid of a concave magnetic driver (magnetic controlled growth rod) and a contralateral passive sliding rod construct with apical control: preliminary report on 17 cases. Spine J.

[7] Thakar C, Kieser DC, Mardare M (2018). Systematic review of the complications associated with magnetically controlled growing rods for the treatment of early onset scoliosis. Eur Spine J.

[8] Thompson GH, Akbarnia BA, Kostial P (2005). Comparison of single and dual growing rod techniques followed through definitive surgery: a preliminary study. Spine.

[9] Polly DW, Ackerman SJ, Schneider K (2016). Cost analysis of magnetically controlled growing rods compared with traditional growing rods for early-onset scoliosis in the US: an integrated health care delivery system perspective. Clinicoecon Outcomes Res.

[10] Wong CKH, Cheung JPY, Cheung PWH (2017). Traditional growing rod versus magnetically controlled growing rod for treatment of early onset scoliosis: cost analysis from implantation till skeletal maturity. J Orthop Surg-Hong.

[11] Charroin C, Abelin-Genevois K, Cunin V (2014). Direct costs associated with the management of progressive early onset scoliosis: estimations based on gold standard technique or with magnetically controlled growing rods. Orthop Traumatol Surg Res.

[12] von Elm E, Altman DG, Egger M (2008). The Strengthening the Reporting of Observational Studies in Epidemiology [STROBE] statement: guidelines for reporting observational studies. Gac Sanit.

[13] Nuvasive (2018) MAGEC noninvasive growth modulation. Accessed 02 Nov 2018

[14] Nash CL, Moe JH (1969). A study of vertebral rotation. J Bone Joint Surg.

[15] Kwan KYH, Alanay A, Yazici M (2017). Unplanned reoperations in magnetically controlled growing rod surgery for early onset scoliosis with a minimum of two-year follow-up. Spine.

[16] Hosseini P, Pawelek J, Mundis GM (2016). Magnetically controlled growing rods for early-onset scoliosis: a multicenter study of 23 cases with minimum 2 years follow-up. Spine.

[17] Lebon J, Batailler C, Wargny M (2017). Magnetically controlled growing rod in early onset scoliosis: a 30-case multicenter study. Eur Spine J.

[18] Keskinen H, Helenius I, Nnadi C (2016). Preliminary comparison of primary and conversion surgery with magnetically controlled growing rods in children with early onset scoliosis. Eur Spine J.

[19] Canavese F, Dimeglio A (2013). Normal and abnormal spine and thoracic cage development. World J Orthop.

[20] Wijdicks SP, Tromp IN, Yazici M, Kempen DH, Castelein RM, Kruyt MC (2018). A comparison of growth among growth friendly systems for scoliosis: a systematic review. Spine J.

[21] Sankar WN, Skaggs DL, Yazici M (2011). Lengthening of dual growing rods and the law of diminishing returns. Spine.

[22] Cheung JPY, Yiu KKL, Samartzis D (2018). Rod lengthening with the magnetically controlled growing rod: factors influencing rod slippage and reduced gains during distractions. Spine.

[23] Akbarnia BA, Marks DS, Boachie-Adjei O (2005). Dual growing rod technique for the treatment of progressive early-onset scoliosis: a multicenter study. Spine.

[24] Teoh KH, Winson DM, James SH (2016). Magnetic controlled growing rods for early-onset scoliosis: a 4-year follow-up. Spine J.

[25] Subramanian T, Ahmad A, Mardare DM (2018). A six-year observational study of 31 children with early-onset scoliosis treated using magnetically controlled growing rods with a minimum follow-up of two years. Bone Joint J.

